# Supplementary material for: Multi‐dataset identification of innovative feature genes and molecular mechanisms in keratoconus
Source: J Cell Mol Med. 2024 Sep 19;28(18):e70079. doi: 10.1111/jcmm.70079 (PMC11412914; doi:10.1111/jcmm.70079)
Supplement: Supplementary file 2 — Material S2. [file JCMM-28-e70079-s002.zip › 7.GSEA analysis/5.RNF217-AS1/KEGG_GLYCINE_SERINE_AND_THREONINE_METABOLISM.html]

Details for gene set KEGG\_GLYCINE\_SERINE\_AND\_THREONINE\_METABOLISM[GSEA]

|  || Dataset | RNF217-AS1.group.cls#h\_versus\_l |
| Phenotype | group.cls#h\_versus\_l |
| Upregulated in class | h |
| GeneSet | KEGG\_GLYCINE\_SERINE\_AND\_THREONINE\_METABOLISM |
| Enrichment Score (ES) | 0.22179772 |
| Normalized Enrichment Score (NES) | 0.7502535 |
| Nominal p-value | 0.9437751 |
| FDR q-value | 1.0 |
| FWER p-Value | 1.0 |
Table: GSEA Results Summary

  

Fig 1: Enrichment plot: KEGG\_GLYCINE\_SERINE\_AND\_THREONINE\_METABOLISM      
 Profile of the Running ES Score & Positions of GeneSet Members on the Rank Ordered List

  

| SYMBOL | TITLE | RANK IN GENE LIST | RANK METRIC SCORE | RUNNING ES | CORE ENRICHMENT || 1 | MAOB | na | 1741 | 0.370 | 0.0380 | Yes |
| 2 | ALAS2 | na | 2048 | 0.346 | 0.1115 | Yes |
| 3 | AGXT2 | na | 2884 | 0.296 | 0.1579 | Yes |
| 4 | DMGDH | na | 4454 | 0.236 | 0.1688 | Yes |
| 5 | AOC2 | na | 5673 | 0.193 | 0.1798 | Yes |
| 6 | BHMT | na | 5787 | 0.190 | 0.2218 | Yes |
| 7 | AMT | na | 7860 | 0.131 | 0.1933 | No |
| 8 | GLYCTK | na | 12002 | 0.055 | 0.0871 | No |
| 9 | GNMT | na | 13037 | 0.042 | 0.0673 | No |
| 10 | PIPOX | na | 13470 | 0.037 | 0.0635 | No |
| 11 | SHMT2 | na | 16383 | 0.005 | -0.0192 | No |
| 12 | MAOA | na | 19080 | -0.021 | -0.0920 | No |
| 13 | GAMT | na | 19425 | -0.025 | -0.0960 | No |
| 14 | SRR | na | 19844 | -0.028 | -0.1013 | No |
| 15 | SHMT1 | na | 20561 | -0.036 | -0.1135 | No |
| 16 | ALAS1 | na | 20839 | -0.038 | -0.1124 | No |
| 17 | DAO | na | 21150 | -0.042 | -0.1114 | No |
| 18 | GLDC | na | 22772 | -0.059 | -0.1440 | No |
| 19 | CBS | na | 23765 | -0.072 | -0.1555 | No |
| 20 | GCAT | na | 24144 | -0.077 | -0.1480 | No |
| 21 | CTH | na | 25070 | -0.091 | -0.1529 | No |
| 22 | PHGDH | na | 25164 | -0.093 | -0.1335 | No |
| 23 | GATM | na | 25424 | -0.097 | -0.1178 | No |
| 24 | DLD | na | 25598 | -0.100 | -0.0990 | No |
| 25 | SARDH | na | 27109 | -0.128 | -0.1120 | No |
| 26 | AOC3 | na | 28131 | -0.152 | -0.1053 | No |
| 27 | PSAT1 | na | 28931 | -0.174 | -0.0868 | No |
| 28 | SDS | na | 29501 | -0.191 | -0.0577 | No |
| 29 | AGXT | na | 29817 | -0.201 | -0.0190 | No |
| 30 | PSPH | na | 30975 | -0.241 | 0.0050 | No |
| 31 | CHDH | na | 33705 | -0.433 | 0.0295 | No |
Table: GSEA details [plain text format]

  

Fig 2: KEGG\_GLYCINE\_SERINE\_AND\_THREONINE\_METABOLISM      
 Blue-Pink O' Gram in the Space of the Analyzed GeneSet

  

Fig 3: KEGG\_GLYCINE\_SERINE\_AND\_THREONINE\_METABOLISM: Random ES distribution      
 Gene set null distribution of ES for **KEGG\_GLYCINE\_SERINE\_AND\_THREONINE\_METABOLISM**
